# (1*E*,2*E*)-1,2-Bis[1-(3-chloro­phen­yl)ethyl­idene]hydrazine

**DOI:** 10.1107/S1600536811049725

**Published:** 2011-11-25

**Authors:** Hoong-Kun Fun, Patcharaporn Jansrisewangwong, Chatchanok Karalai, Suchada Chantrapromma

**Affiliations:** a X-ray Crystallography Unit, School of Physics, Universiti Sains Malaysia, 11800 USM, Penang, Malaysia; b Crystal Materials Research Unit, Department of Chemistry, Faculty of Science, Prince of Songkla University, Hat-Yai, Songkhla 90112, Thailand

## Abstract

The title mol­ecule, C_16_H_14_Cl_2_N_2_, lies on an inversion center. The dihedral angle between the symmetry-related benzene rings is 0.02 (11)°. The mean plane of the central C(meth­yl)—C=N—N=C—C(meth­yl) unit forms a dihedral angle of 5.57 (12)° with the symmetry-unique benzene ring.

## Related literature

For background to the biological activity and fluorescent properties of hydrazones, see: Li *et al.* (2009 ▶); Qin *et al.* (2009 ▶). For related structures see: Chantrapromma *et al.* (2010 ▶); Fun *et al.* (2010 ▶, 2011 ▶); Jansrisewangwong *et al.* (2010 ▶); Nilwanna *et al.* (2011 ▶). For standard bond-length data, see: Allen *et al.* (1987 ▶).
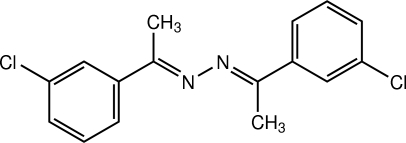


## Experimental

### 

#### Crystal data

C_16_H_14_Cl_2_N_2_
*M* *_r_* = 305.19
Monoclinic, 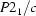

*a* = 10.7796 (18) Å
*b* = 5.2725 (9) Å
*c* = 15.3427 (18) Å
β = 121.540 (8)°
*V* = 743.2 (2) Å^3^
*Z* = 2
Mo *K*α radiation
μ = 0.43 mm^−1^
*T* = 297 K
0.31 × 0.15 × 0.11 mm

#### Data collection

Bruker APEX DUO CCD area-detector diffractometer
Absorption correction: multi-scan (*SADABS*; Bruker, 2009 ▶) *T*_min_ = 0.880, *T*_max_ = 0.957
7616 measured reflections
1970 independent reflections
1469 reflections with *I* > 2σ(*I*)
*R*_int_ = 0.028

#### Refinement

*R*[*F* ^2^ > 2σ(*F* ^2^)] = 0.051
*wR*(*F* ^2^) = 0.180
*S* = 1.09
1970 reflections
92 parameters
H-atom parameters constrained
Δρ_max_ = 0.46 e Å^−3^
Δρ_min_ = −0.41 e Å^−3^

### 

Data collection: *APEX2* (Bruker, 2009 ▶); cell refinement: *SAINT* (Bruker, 2009 ▶); data reduction: *SAINT*; program(s) used to solve structure: *SHELXTL* (Sheldrick, 2008 ▶); program(s) used to refine structure: *SHELXTL*; molecular graphics: *SHELXTL*; software used to prepare material for publication: *SHELXTL* and *PLATON* (Spek, 2009 ▶).

## Supplementary Material

Crystal structure: contains datablock(s) global, I. DOI: 10.1107/S1600536811049725/lh5380sup1.cif

Structure factors: contains datablock(s) I. DOI: 10.1107/S1600536811049725/lh5380Isup2.hkl

Supplementary material file. DOI: 10.1107/S1600536811049725/lh5380Isup3.cml

Additional supplementary materials:  crystallographic information; 3D view; checkCIF report

## Figures and Tables

**Fig. 1.**
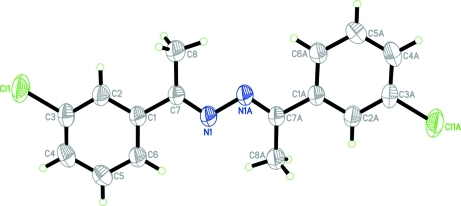
The molecular structure of (I), showing 50% probability displacement ellipsoids. Atoms with suffix A were generated by symmetry code -x, 1-y, 2-z.

**Fig. 2.**
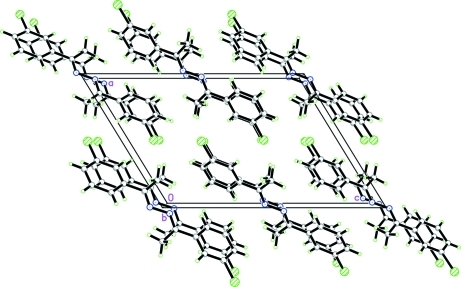
The crystal packing of (I). No clasical hydrogen bonds nor weak interactions are observed in the crystal structure

## supplementary crystallographic information

### Comment

Due to the interesting applications of hydrazones with respect to their antibacterial, antiviral and antioxidant (Li *et al.*, 2009) as well as fluorescent properties (Qin *et al.*, 2009), we have synthesized a series of hydrazones in order to study these activities and have reported some of these crystal structures (Chantrapromma *et al.*, 2010; Fun *et al.*, 2010,2011; Jansrisewangwong *et al.*, 2010; Nilwanna *et al.*, 2011). As part of our on-going research on the medicinal chemistry of hydrazones, the title compound (I) was synthesized and its biological activities will be reported elsewhere. However, it does not possess fluorescent property.

The molecular structure of (I) is shown in Fig. 1. The asymmetric unit contains half a molecule and the complete molecule is generated by a crystallographic inversion center at -x, 1-y, 2-z. The molecule exists in an *E*,*E* configuration with respect to the two ethylidene C═N bonds [1.279 (3) Å] and the torsion angle N1A–N1–C7–C1 = 179.8 (2)°. The molecule is essentially planar with the dihedral angle between the two benzene rings of 0.02 (11)°. The diethylidenehydrazine moiety (C7/C8/N1/N1A/C7A/C8A) is planar with the *r.m.s* of 0.0015 (2) Å. This central C(methyl)—C═N—N═C—C(methyl) mean plane makes the dihedral angle of 5.57 (12)° with the adjacent benzene rings. The bond distances are within the normal range (Allen *et al.*, 1987) and are comparable with the related structures (Chantrapromma *et al.*, 2010; Fun *et al.*, 2010; 2011; Jansrisewangwong *et al.*, 2010; Nilwanna *et al.*, 2011).

Although no clasical hydrogen bonds or weak interactions were observed in the crystal structure, the crystal packing is shown in Fig. 2.

### Experimental

The title compound (I) was synthesized by mixing a solution (1:2 molar ratio) of hydrazine hydrate (0.10 ml, 2 mmol) and 3-chloroacetophenone (0.50 ml, 4 mmol) in ethanol (20 ml). The resulting solution was refluxed for 7 h, yielding the yellow crystalline solid. The resultant solid was filtered off and washed with methanol. Yellow block-shaped single crystals of the title compound suitable for *x*-ray structure determination were recrystalized from acetone by slow evaporation of the solvent at room temperature over several days, Mp. 356-358 K.

### Refinement

All H atoms were positioned geometrically and allowed to ride on their parent atoms, with d(C-H) = 0.93 Å for aromatic and 0.96 Å for CH_3_ atoms. The *U*_iso_ values were constrained to be 1.5*U*_eq_ of the carrier atom for methyl H atoms and 1.2*U*_eq_ for the remaining H atoms. A rotating group model was used for the methyl groups. The highest residual electron density peak is located at 1.92 Å from H8B and the deepest hole is located at 0.70 Å from Cl1.

### Crystal data

**Table:**

C_16_H_14_Cl_2_N_2_	*F*(000) = 316
*M**_r_* = 305.19	*D*_x_ = 1.364 Mg m^−^^3^
Monoclinic, *P*2_1_/*c*	Melting point = 356–358 K
Hall symbol: -P 2ybc	Mo *K*α radiation, λ = 0.71073 Å
*a* = 10.7796 (18) Å	Cell parameters from 1970 reflections
*b* = 5.2725 (9) Å	θ = 2.2–29.0°
*c* = 15.3427 (18) Å	µ = 0.43 mm^−^^1^
β = 121.540 (8)°	*T* = 297 K
*V* = 743.2 (2) Å^3^	Block, yellow
*Z* = 2	0.31 × 0.15 × 0.11 mm

### Data collection

**Table:**

Bruker APEX DUO CCD area-detector diffractometer	1970 independent reflections
Radiation source: sealed tube	1469 reflections with *I* > 2σ(*I*)
graphite	*R*_int_ = 0.028
φ and ω scans	θ_max_ = 29.0°, θ_min_ = 2.2°
Absorption correction: multi-scan (*SADABS*; Bruker, 2009)	*h* = −14→14
*T*_min_ = 0.880, *T*_max_ = 0.957	*k* = −7→6
7616 measured reflections	*l* = −20→20

### Refinement

**Table:**

Refinement on *F*^2^	Primary atom site location: structure-invariant direct methods
Least-squares matrix: full	Secondary atom site location: difference Fourier map
*R*[*F*^2^ > 2σ(*F*^2^)] = 0.051	Hydrogen site location: inferred from neighbouring sites
*wR*(*F*^2^) = 0.180	H-atom parameters constrained
*S* = 1.09	*w* = 1/[σ^2^(*F*_o_^2^) + (0.0951*P*)^2^ + 0.2508*P*] where *P* = (*F*_o_^2^ + 2*F*_c_^2^)/3
1970 reflections	(Δ/σ)_max_ = 0.001
92 parameters	Δρ_max_ = 0.46 e Å^−^^3^
0 restraints	Δρ_min_ = −0.41 e Å^−^^3^

### Special details

**Table:**

Geometry. All esds (except the esd in the dihedral angle between two l.s. planes) are estimated using the full covariance matrix. The cell esds are taken into account individually in the estimation of esds in distances, angles and torsion angles; correlations between esds in cell parameters are only used when they are defined by crystal symmetry. An approximate (isotropic) treatment of cell esds is used for estimating esds involving l.s. planes.
Refinement. Refinement of F^2^ against ALL reflections. The weighted R-factor wR and goodness of fit S are based on F^2^, conventional R-factors R are based on F, with F set to zero for negative F^2^. The threshold expression of F^2^ > 2sigma(F^2^) is used only for calculating R-factors(gt) etc. and is not relevant to the choice of reflections for refinement. R-factors based on F^2^ are statistically about twice as large as those based on F, and R- factors based on ALL data will be even larger.

### Fractional atomic coordinates and isotropic or equivalent isotropic displacement parameters (Å^2^)

**Table:**

	*x*	*y*	*z*	*U*_iso_*/*U*_eq_	
Cl1	0.49474 (7)	0.23824 (14)	0.85934 (6)	0.0708 (3)	
N1	0.0247 (2)	0.5365 (4)	0.96753 (14)	0.0543 (5)	
C1	0.1907 (2)	0.4809 (4)	0.91400 (14)	0.0413 (4)	
C2	0.3036 (2)	0.3431 (4)	0.91724 (16)	0.0460 (5)	
H2A	0.3438	0.2045	0.9607	0.055*	
C3	0.3550 (2)	0.4148 (4)	0.85517 (16)	0.0478 (5)	
C4	0.2988 (2)	0.6198 (5)	0.78983 (17)	0.0527 (6)	
H4A	0.3353	0.6655	0.7490	0.063*	
C5	0.1869 (3)	0.7552 (4)	0.78659 (19)	0.0535 (6)	
H5A	0.1476	0.8939	0.7430	0.064*	
C6	0.1324 (2)	0.6875 (4)	0.84733 (16)	0.0468 (5)	
H6A	0.0565	0.7801	0.8438	0.056*	
C7	0.1350 (2)	0.4107 (4)	0.98120 (15)	0.0426 (4)	
C8	0.2087 (3)	0.2056 (6)	1.0585 (2)	0.0711 (8)	
H8A	0.1613	0.1850	1.0964	0.107*	
H8B	0.3088	0.2501	1.1045	0.107*	
H8C	0.2034	0.0496	1.0245	0.107*	

### Atomic displacement parameters (Å^2^)

**Table:**

	*U*^11^	*U*^22^	*U*^33^	*U*^12^	*U*^13^	*U*^23^
Cl1	0.0653 (4)	0.0831 (5)	0.0902 (5)	0.0089 (3)	0.0590 (4)	−0.0068 (3)
N1	0.0601 (11)	0.0663 (12)	0.0567 (10)	0.0214 (9)	0.0445 (9)	0.0206 (9)
C1	0.0438 (10)	0.0463 (10)	0.0414 (9)	0.0004 (8)	0.0276 (8)	−0.0015 (8)
C2	0.0470 (10)	0.0503 (11)	0.0487 (10)	0.0038 (9)	0.0306 (9)	−0.0020 (9)
C3	0.0459 (10)	0.0571 (13)	0.0518 (11)	−0.0052 (9)	0.0335 (9)	−0.0125 (9)
C4	0.0611 (13)	0.0609 (14)	0.0530 (11)	−0.0129 (11)	0.0415 (11)	−0.0081 (10)
C5	0.0604 (13)	0.0577 (14)	0.0507 (12)	0.0002 (10)	0.0347 (11)	0.0068 (9)
C6	0.0466 (10)	0.0547 (12)	0.0473 (10)	0.0046 (9)	0.0302 (9)	0.0048 (9)
C7	0.0471 (10)	0.0470 (11)	0.0435 (9)	0.0054 (8)	0.0305 (8)	0.0023 (8)
C8	0.0766 (17)	0.0858 (19)	0.0761 (16)	0.0374 (15)	0.0574 (15)	0.0370 (15)

### Geometric parameters (Å, °)

**Table:**

Cl1—C3	1.743 (2)	C4—C5	1.380 (3)
N1—C7	1.279 (3)	C4—H4A	0.9300
N1—N1^i^	1.406 (3)	C5—C6	1.383 (3)
C1—C2	1.395 (3)	C5—H5A	0.9300
C1—C6	1.399 (3)	C6—H6A	0.9300
C1—C7	1.486 (3)	C7—C8	1.491 (3)
C2—C3	1.382 (3)	C8—H8A	0.9600
C2—H2A	0.9300	C8—H8B	0.9600
C3—C4	1.380 (3)	C8—H8C	0.9600
			
C7—N1—N1^i^	113.9 (2)	C4—C5—H5A	119.6
C2—C1—C6	118.78 (18)	C6—C5—H5A	119.6
C2—C1—C7	120.47 (19)	C5—C6—C1	120.5 (2)
C6—C1—C7	120.74 (18)	C5—C6—H6A	119.8
C3—C2—C1	119.3 (2)	C1—C6—H6A	119.8
C3—C2—H2A	120.3	N1—C7—C1	115.82 (18)
C1—C2—H2A	120.3	N1—C7—C8	124.68 (19)
C4—C3—C2	122.2 (2)	C1—C7—C8	119.49 (18)
C4—C3—Cl1	119.20 (16)	C7—C8—H8A	109.5
C2—C3—Cl1	118.63 (18)	C7—C8—H8B	109.5
C5—C4—C3	118.4 (2)	H8A—C8—H8B	109.5
C5—C4—H4A	120.8	C7—C8—H8C	109.5
C3—C4—H4A	120.8	H8A—C8—H8C	109.5
C4—C5—C6	120.9 (2)	H8B—C8—H8C	109.5
			
C6—C1—C2—C3	0.3 (3)	C2—C1—C6—C5	−0.6 (3)
C7—C1—C2—C3	−178.95 (19)	C7—C1—C6—C5	178.6 (2)
C1—C2—C3—C4	0.2 (3)	N1^i^—N1—C7—C1	179.8 (2)
C1—C2—C3—Cl1	−179.32 (16)	N1^i^—N1—C7—C8	−0.5 (4)
C2—C3—C4—C5	−0.4 (3)	C2—C1—C7—N1	−175.2 (2)
Cl1—C3—C4—C5	179.15 (17)	C6—C1—C7—N1	5.6 (3)
C3—C4—C5—C6	0.0 (4)	C2—C1—C7—C8	5.1 (3)
C4—C5—C6—C1	0.5 (4)	C6—C1—C7—C8	−174.1 (2)

Symmetry codes: (i) −*x*, −*y*+1, −*z*+2.
